# A simple nomogram for assessing the risk of IgA vasculitis nephritis in IgA vasculitis Asian pediatric patients

**DOI:** 10.1038/s41598-022-20369-3

**Published:** 2022-10-07

**Authors:** Yuna Bi, Wei Quan, Wei Hao, Rui Sun, Liwen Li, Chunping Jiang, Lingling Tian, Lin Liu, Jie Liu, Xiaozhong Li, Tao Li

**Affiliations:** 1 Department of Nephrology and Immunology, Children’s Hospital of Soochow University, 303 Jingde Road, Suzhou, 215003 Jiangsu China; 2 Department of Pediatrics, Shandong Provincial Hospital Affiliated to Shandong First Medical University, Jinan, China; 3 Cheeloo College of Medicine, Shandong University, Jinan, China; 4 Department of Pediatrics, Shandong Provincial Maternal and Child Health Care Hospital, Jinan, China; 5 Department of Infectious Diseases, Shandong Provincial Hospital Affiliated to Shandong First Medical University, 324#, Jing 5 Road, Jinan, 250021 China

**Keywords:** Nephritis, Predictive markers

## Abstract

A nomogram for assessing the risk of IgA vasculitis nephritis (originally termed Henoch–Schönlein purpura nephritis, HSPN) in IgA vasculitis (originally termed Henoch–Schönlein purpura, HSP) pediatric patients can effectively improve early diagnosis and prognosis of IgA vasculitis nephritis. However, currently, no nomogram is available. 246 IgA vasculitis and 142 IgA vasculitis nephritis Asian pediatric patients confirmed by renal biopsy were enrolled. Univariate and multivariate logistic regressions were performed to identify the independent risk factors and construct a series of predictive models. The receiver operating characteristic curve, calibration plot, decision curve analysis, net reclassification index and integrated discrimination index were used to screen the best model. Stratification analysis was applied to optimize model’s clinical utility. An external validation set was introduced to verify the predictive efficiency. The final predictive model was converted to nomogram for visual use. We identified age, duration of rash (Dor), D-dimer and IgG as independent risk factors and constructed four models as follows: AIDD (Age + IgG + Dor + D-dimer), AIDi (Age + IgG + D-dimer), AIDo (Age + IgG + Dor) and ADD (Age + Dor + D-dimer), which achieved the receiver operator characteristic curve (AUROC) of 0.931, 0.920, 0.856 and 0.907, respectively. Finally, AIDi model with an AUROC of 0.956 and 0.897 in internal and external validating sets was proposed as a novel predictive model. In stratification analysis by gender and histological grade, the AUROC of AIDi was 0.949 in female, 0.926 in male, 0.933 in mild histological grades and 0.939 in severe histological grades, respectively. AIDi nomogram is an effective and visual tool for assessing the risk of nephritis in IgA vasculitis Asian pediatric patients, regardless of IgA vasculitis nephritis histological grades and gender.

## Introduction

IgA vasculitis nephritis (originally termed Henoch–Schönlein purpura nephritis), a serious complication of IgA vasculitis (originally termed Henoch–Schönlein purpura), can occur in up to 50% of pediatric patients during the course of IgA vasculitis^1^. IgA vasculitis nephritis is characterized by microscopic hematuria and/or proteinuria and a few started with acute glomerulonephritis or nephrotic syndrome. Although the majority of IgA vasculitis nephritis pediatric patients have a good prognosis, a small number of children continue to progress to chronic kidney disease. IgA vasculitis nephritis has become one of the important causes of end-stage kidney disease in children^2^. Therefore, as the kidney involvement is the most important conditioning prognosis of IgA vasculitis early prediction and timely treatment is essential for prognosis of IgA vasculitis nephritis, and it can also improve follow-up management of IgA vasculitis in children^3^.

At present, many risk factors from clinical characteristics have been verified to be closely related with the occurrence of IgA vasculitis nephritis in pediatric patients with IgA vasculitis^4,5^. Persistent or recurrent purpura, severe abdominal symptoms and an older age were proved as the most significant risk factors for the IgA vasculitis nephritis^4,6^. It was believed that skin purpura outside the lower limbs and persistent purpura or recurrent purpura specially lasting more than 1 month were related to the occurrence of IgA vasculitis nephritis^4,5,7^. Additionally, IgA vasculitis is in the state of systemic immune inflammation characterized by involvements of skin, joint and gastrointestinal tract involvement, lots of immune indicators changing with ongoing immune inflammation process also have the potential to predict the development of IgA vasculitis nephritis. The disorders of immunoglobulin in IgA vasculitis nephritis patients, including IgG, IgM, IgA and IgE, were very common and significant^8,9^. Some studies suggested that abnormality of coagulation and fibrinolytic system also were associated with various renal diseases in children, including IgA vasculitis nephritis^10^. Increasing level of D-dimer, indicated the enhancement of secondary fibrinolytic activity, can be the potential risk factor for kidney damage in IgA vasculitis patients^11^. Xu et al. reported that red cell distribution width (RDW) from peripheral complete blood cell count examination was an independent predictor of IgA vasculitis nephritis and its levels greater than 13.25 were useful in the predicting the presence of crescents on histopathology^12^. Hence, it is promising to screen and identify valuable risk factors and construct a combined predictive model for IgA vasculitis nephritis. However, currently, the predictive models are still relatively limited, and the models with higher predictive efficiency still need to be further explored.

In this study, 388 Asian participants including IgA vasculitis cases (n = 246) and IgA vasculitis nephritis cases (n = 142) were enrolled and assigned to the training set (n = 138), internal validation set (n = 128) and external validating set (n = 122). All data of clinical characteristics, immune and inflammatory indicators and coagulation and fibrinolytic system indicators, which including peripheral complete blood count (CBC) with differential, immune globulins, complement serum level, fibrinogen and D-dimer from the patient’s routine lab examination. A novel predictive model for IgA vasculitis nephritis based on these data was constructed and assessed the predictive efficiency by an external validating set. The final predictive model was converted to nomogram for visual use. This predictive nomogram can help clinicians make early diagnosis, timely treatment and ultimately improve the prognosis of the IgA vasculitis nephritis of Asian pediatric patients.

## Results

### Screening of candidate predictors for the risk of development of IgA vasculitis nephritis

The characteristics of the all participants are summarized in Table 1 and Table S1. This study includes 147 males and 119 females with the median age being 8 years, ranging from 2 to 15 years. In the training set, the IgA vasculitis nephritis group showed significantly lower platelets (PLT) count (319.5 (280.3, 343.0) vs. 355.0 (287.3, 413.0), *p* = 0.019), C-reactive protein (CRP) (0.75 (0.25, 1.38) vs. 3.52 (1.30, 10.71), *p* = 0.0001), (D-dimer) (0.32 (0.23, 0.51) vs. 1.88 (0.90, 3.55), *p* = 0.0001), IgG (9.30 (6.11, 9.87) vs. 10.25 (8.69, 12.38), *p* = 0.0001) and complement 3 (C3) (1.14 ± 0.13 vs. 1.22 ± 0.22, *p* = 0.033). Other variables, such as the proportion of patients with rash more than a month (58.3% vs. 26.7%, *p* = 0.001), age (10.50 (8.00, 12.00) vs. 7.00 (5.25, 9.00), *p* = 0.0001), weight (43.00 (31.00, 55.75) vs. 26.50 (20.50, 37.00), *p* = 0.0001), mean corpuscular volume (MCV) (87.35 (84.25, 89.82) vs. 84.10 (81.05, 86.38), *p* = 0.0001) and red cell distribution width (RDW) (41.75 (38.85, 44.85) vs. 39.80 (38.00, 41.38), *p* = 0.001) were significantly higher in the IgA vasculitis nephritis group than the IgA vasculitis group. These results showed that there were many significant differences between IgA vasculitis and IgA vasculitis nephritis group, which indicated a promising strategy for screening the valuable predictors for evaluating the risk of IgA vasculitis nephritis. No significant difference in gender, white blood cell (WBC), neutrophil (NEU), lymphocytes (LYM), monocyte (MON), red blood cell (RBC) count, hemoglobin (HGB), platelet distribution width (PDW), mean platelet volume (MPV), activated partial thromboplastin time (APTT), Fibrinogen (Fib), IgA, IgM and complement 4 (C4). This followed the similar trend as in the validating set and indicated that these two sets were well balanced in baseline characteristics.

**Table 1:** Characteristics of the pediatric patients with IgA vasculitis and IgA vasculitis nephritis.

Variables (n = 24)	Training set (n = 138)			Internal validation set (n = 128)		
	IgA vasculitis (n = 90)	IgA vasculitis nephritis (n = 48)	*p*	IgA vasculitis (n = 84)	IgA vasculitis nephritis (n = 44)	*p*
**Gender**						
Female, n (%)	41 (45.6)	21 (43.8)	0.981	33 (39.3)	24 (54.5)	0.144
Male, n (%)	49 (54.4)	27 (56.2)		51 (60.7)	20 (45.5)	
Age (IQR) (years)	7.00 (5.25, 9.00)	10.50 (8.00, 12.00)	0.0001*	6.00 (5.00, 7.25)	9.00 (7.00, 11.25)	0.0001*
Weigh (IQR) (kg)	26.50 (20.50, 37.00)	43.00 (31.00, 55.75)	0.0001*	23.25 (19.00, 29.00)	38.50 (29.75, 47.88)	0.0001*
**Duration of rash**^**#**^						
Within a month, n (%)	66 (73.3)	20 (41.7)	0.001*	70 (83.3)	30 (68.2)	0.081
More than a month, n (%)	24 (26.7)	28 (58.3)		14 (16.7)	14 (31.8)	
WBC (IQR) (10^9^/L)	10.09 (8.33, 12.61)	10.71 (7.84, 13.09)	0.853	9.54 (7.56, 12.54)	11.27 (6.31, 16.47)	0.299
NEU (IQR) (10^9^/L)	5.98 (3.76, 8.99)	6.30 (4.34, 9.35)	0.495	5.69 (4.29, 7.86)	6.86 (4.15, 10.14)	0.203
LYM (IQR) (10^9^/L)	2.94 (2.35, 4.06)	3.08 (2.41, 3.71)	0.977	2.86 (2.18, 3.72)	3.04 (2.50, 4.97)	0.056
MON (IQR) (10^9^/L)	0.63 (0.43, 0.88)	0.66 (0.40, 0.82)	0.818	0.60 (0.39, 0.77)	0.70 (0.44, 0.95)	0.103
PLT (IQR) (10^9^/L)	355.0 (287.3, 413.0)	319.5 (280.3, 343.0)	0.019*	347.0 (290.3, 403.5)	303.0 (247.5, 346.0)	0.001*
RBC (10^12^/L)	4.71 (4.42, 5.05)	4.78 (4.41, 4.98)	0.907	4.78 (4.54, 4.94)	4.82 (4.56, 5.22)	0.177
HGB (g/L)	133.7 ± 12.54	136.99 ± 15.89	0.188	134.0 (128.0, 140.3)	136.5 (132.0, 144.3)	0.094
MCV (fL)	84.10 (81.05, 86.38)	87.35 (84.25, 89.82)	0.0001*	82.85 (80.77, 85.43)	86.35 (83.38, 89.48)	0.0001*
RDW (fL)	39.80 (38.00, 41.38)	41.75 (38.85, 44.85)	0.001*	39.13 ± 2.61	41.21 ± 4.37	0.001*
PDW (IQR) (fL)	10.40 (9.20, 11.70)	10.40 (9.67, 11.93)	0.363	10.55 (9.70, 12.72)	10.75 (9.78, 12.75)	0.505
MPV (fL)	9.40 (8.65, 10.00)	9.40 (9.20, 10.20)	0.099	9.40 (8.80, 10.05)	9.75 (8.80, 10.60	0.182
CRP (IQR) (mg/L)	3.52 (1.30, 10.71)	0.75 (0.25, 1.38)	0.0001*	2.78 (1.07, 8.27)	0.604 (0.21, 1.38)	0.0001*
D-dimer (IQR) (mg/L)	1.88 (0.90, 3.55)	0.32 (0.23, 0.51)	0.0001*	1.41 (0.70, 2.52)	0.36 (0.20, 0.62)	0.0001*
APTT (s)	33.12 ± 6.56	32.33 ± 9.38	0.568	33.97 ± 5.91	32.56 ± 5.52	0.193
Fib (g/L)	3.37 (2.73, 3.90)	3.24 (2.90, 3.76)	0.461	3.34 (2.94, 3.77)	3.15 (2.33, 3.76)	0.243
IgA (IQR) (g/L)	2.29 (1.79, 2.68)	2.05 (1.78, 2.16)	0.166	2.22 (1.65, 2.88)	2.05 (1.62, 2.11)	0.022*
IgM (IQR) (g/L)	1.15 (0.89, 1.42)	1.34 (0.84, 1.54)	0.137	1.02 (0.83, 1.35)	1.50 (1.00, 1.64)	0.001*
IgG (IQR) (g/L)	10.25 (8.69, 12.38)	9.30 (6.11, 9.87)	0.0001*	10.10 (8.70, 12.35)	7.64 (5.12, 9.87)	0.0001*
C3 (g/L)	1.22 ± 0.22	1.14 ± 0.13	0.033*	1.21 (1.07, 1.35)	1.15 (1.08, 1.28)	0.251
C4 (g/L)	0.25 (0.21, 0.30)	0.23 (0.21, 0.26)	0.071	0.24 (0.21, 0.31)	0.23 (0.20, 0.26)	0.146
**Histological grade**						
I, n (%)	–	4 (8.3)	–	–	5 (11.4)	–
IIa, n (%)	–	11 (22.9)	–	–	8 (18.1)	–
IIb and above, n (%)	–	33 (68.8)	–	–	31 (70.5)	–

*p < 0.05 for significance.

^#^Means the time of rash recurrent or persistent.

*WBC* white blood cell, *NEU* neutrophil, *LYM* lymphocyte, *MON* monocyte, *PLT* platelet, *RBC* red blood cell, *HGB* hemoglobin, *MCV* mean corpuscular volume, *RDW* red cell distribution width, *PDW* platelet distribution width, *MPV* mean platelet volume, *CRP* c-reaction protein, *APTT* activated partial thromboplastin time, *Fib*. fibrinogen, *IQR* interquartile range.

### Candidate predictors selected from training set and predictive efficiency assessed

These variables with significant differences between IgA vasculitis and IgA vasculitis nephritis group were further screened as valuable candidate predictors. All candidate variables were analyzed by a univariate logistic analysis and further assessed by multivariate logistic regression analysis. As shown in Table 2, a total of 8 variables (age, weigh, Dor, PLT count, CRP, D-dimer, IgG and C3) were selected as determined by statistically significant from the original set of 24 variables in the training set using univariate logistic analysis. Weight was excluded for tighter linkage with age. D-dimer with a markedly skewed distribution was square root transformed to decrease the impact of extreme observations. These 7 candidate predictors were further assessed using the multivariate logistic regression analysis. Finally, Age (odds ratio [OR]: 1.297, 95% confidence interval [CI]: 1.036–1.663, *p* = 0.029), Dor (OR: 3.606, 95% CI: 1.127–1.285, *p* = 0.036), D-dimer (OR: 0.063, 95% CI: 0.010–0.284, *p* = 0.001) and IgG (OR: 0.648, 95% CI: 0.478–0.828, *p* = 0.002) were identified as independent risk factors associated with development of IgA vasculitis nephritis (Table 2).

**Table 2:** Univariate and multivariate analysis of risk factors for nephritis in IgA vasculitis in training set.

Variables	IgA vasculitis vs. IgA vasculitis nephritis in training set					
	Univariate			Multivariate		
	OR	95% CI	*p*	OR	95% CI	*p*
Age (years)	1.477	(1.275, 1.739)	0.0001*	1.297	(1.036, 1.663)	0.029*
Weight (kg)	1.069	(1.041, 1.103)	0.0001*	–	–	
Duration of rash^#^	3.850	(1.855, 8.189)	0.0001*	3.606	(1.127, 1.285)	0.036*
PLT count (10^9^/L)	0.996	(0.991, 1.000)	0.037*	0.995	(0.988, 1.002)	0.184
CRP (mg/L)	0.701	(0.558, 0.835)	0.0001*	0.889	(0.703, 1.022)	0.213
D-dimer (mg/L)^§^	0.028	(0.007, 0.093)	0.0001*	0.063	(0.010, 0.284)	0.001*
IgG (g/L)	0.716	(0.607, 0.829)	0.0001*	0.648	(0.478, 0.828)	0.002*
C3 (g/L)	0.124	(0.016, 0.815)	0.036*	0.586	(0.009, 1.285)	0.795
C4 (g/L)	0.006	(0.000, 0.644)	0.042*	0.001	(0.000, 6.352)	0.157

*p < 0.05 for significance.

^#^Means the time of rash recurrent or persistent.

^§^The result is the square root of the D-dimer.

*PLT* platelet, *CRP* C-reaction protein, *CI* confidence interval, *OR* odds ratio.

**Figure 1:**
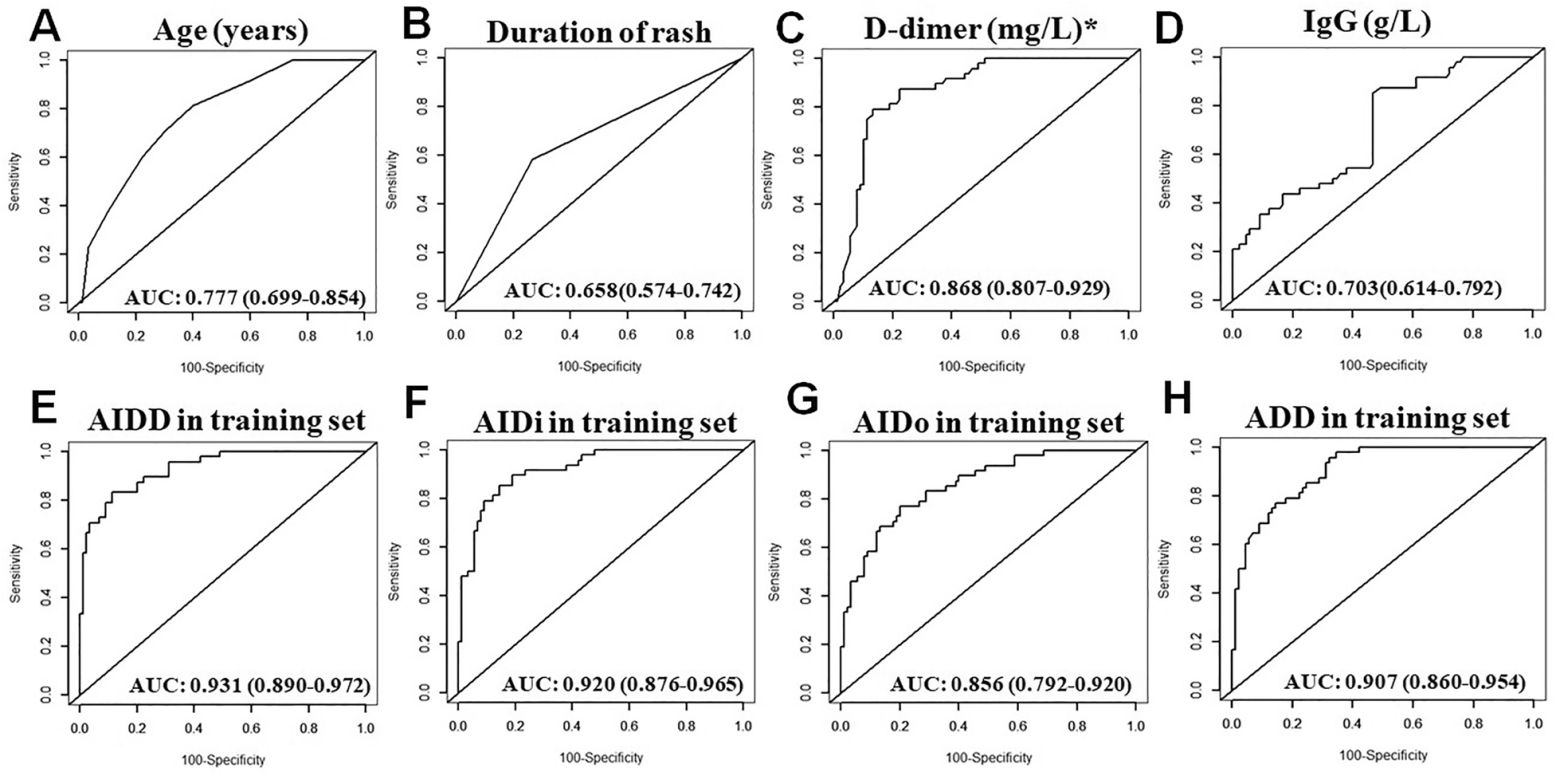
Receiver operating characteristic curve analysis of 4 independent risk factors and 4 combined models for predicting the risk of IgA vasculitis nephritis in training set. AUROC of the age (**A**), duration of rash (**B**), D-dimer (**C**), IgG (**D**), AIDD (**E**), AIDi (**F**), AIDo (**G**) and ADD (**H**). *Based on the square root of the D-dimer level.

ROC analysis of these four risk factors for predicting the development of nephritis from IgA vasculitis were plotted in Fig. 1A–D with AUROC of 0.777 (age, 95% CI: 0.699–0.854), 0.658 (Dor, 95% CI: 0.574–0.742), 0.868 (D-dimer, 95% CI: 0.807–0.929) and 0.703 (IgG, 95% CI: 0.614–0.792), respectively. Among these predictors, the best predictive efficiency was obtained from D-dimer (Fig. 1C). In addition, it was reported that some common immune-inflammation index, such as NEU-to-LYM (NLR), MON-to-LYM (MLR), PLT-to-LYM (PLR) and systemic immune-inflammation index (SII), can also reflect the inflammation level and predict the occurrence of nephritis^13,14^. We also evaluated this index performance in predicting the risk of IgA vasculitis nephritis. As shown in Fig. S1A–D, these index were inefficient for predicting the development of nephritis from IgA vasculitis with an AUROC of only 0.532 (NLR, 95% CI: 0.432–0.632), 0.535 (MLR, 95% CI: 0.434–0.636), 0.535 (PLR, 95% CI: 0.438–0.633) and 0.508 (SII, 95% CI: 0.407–0.608), respectively.

### AIDi was proposed as a novel model for predicting the development of IgA vasculitis nephritis

Four fitted and simplified predictive models, named as AIDD (including Age, Dor, IgG, D-dimer), AIDi (including Age, IgG, D-dimer), AIDo (including Age, Dor, IgG) and ADD (including Age, Dor, D-dimer), were constructed on the basis of these 4 independent risk factors identified in multivariate analysis of the training set (n = 138). In the training set, the AUROC of these 4 predictive models showed a good discrimination in distinguishing nephritis from IgA vasculitis patients (AIDD: 0.931, 95% CI: 0.890–0.972; AIDi: 0.920, 95% CI: 0.876–0.965, AIDo: 0.856, 95% CI: 0.792–0.920 and ADD: 0.907, 95% CI: 0.860–0.954), respectively (Fig. 1E–H). Similar results were also obtained from the internal validating set (Fig. S2–D).

**Figure 2:**
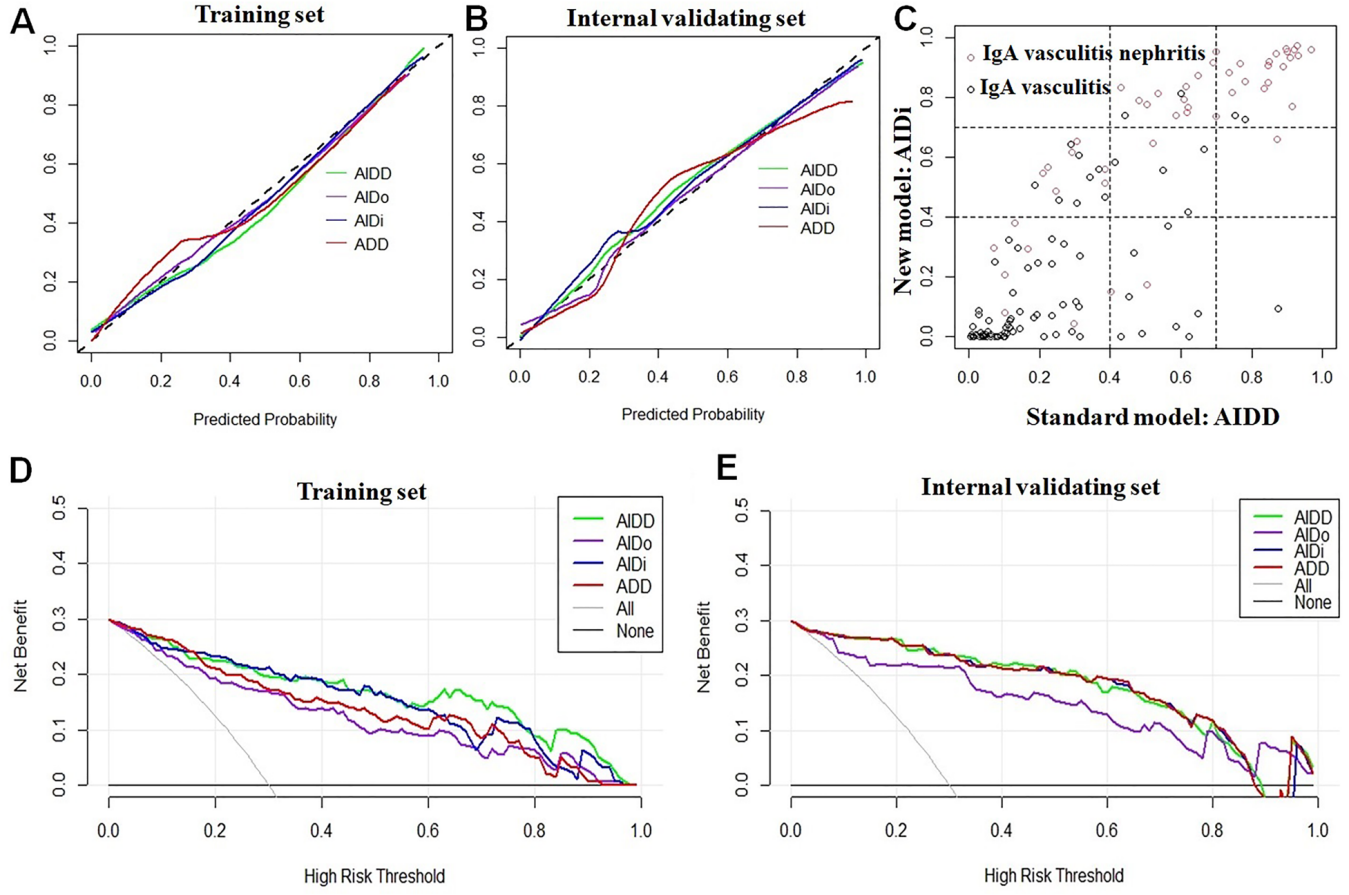
Model calibration and discrimination of 4 combined model for predicting the risk of IgA vasculitis nephritis. Calibration plot (**A**,**B**), net reclassification index (**C**) and decision curve analysis (**D**,**E**). IgA vasculitis nephritis.

The calibration plot for probability of these 4 models showed good consistency between the prediction and actual observation in training set and internal validating set (Fig. 2A,B). However, the model ADD may not predict well for the lower risk of IgA vasculitis nephritis patients. In addition, DCA were also plotted to assess the net benefit of these 4 models. The results showed that model AIDD and AIDo had the highest net benefit in training set and internal validating set (Fig. 2D,E). Moreover, the NRI and IDI were preformed to evaluate whether removing Dor could improve the efficiency of the model AIDD. As shown in Fig. 2C and Table 3, the probability of nephritis occurring in IgA vasculitis was grouped into three groups: low-risk group (< 40%), medium-risk group (40–70%) and high-risk group (> 70%). The NRI for removing Dor from the AIDD model was 0.301 (*p* = 0.018) and the IDI was 0.168 (*p* < 0.001). The results indicated a positive and significant reclassification effect for the predicting the high-risk of IgA vasculitis nephritis for the model AIDi. Finally, model AIDi, with higher predictive efficiency of high-risk of IgA vasculitis nephritis and fewer predictors, was proposed as a novel predictive model for predicting the risk of IgA vasculitis nephritis.

**Table 3:** The predictive efficiency of AIDD and AIDo was evaluated by NRI and IDI in training set.

	Estimate	SE	95% CI	*p*
NRI	0.301	0.128	(0.062, 0.569)	0.018*
NRI+	0.313	0.110	(0.089, 0.517)	0.005*
NRI−	− 0.011	0.043	(− 0.069, 0.095)	0.795
IDI	0.168	–	(0.107, − 0.230)	0.0001*

*p < 0.05 for significance.

*NRI* net reclassification index, *IDI* integrated discrimination index, *CI* confidence interval, *SE* standard error.

**Figure 3:**
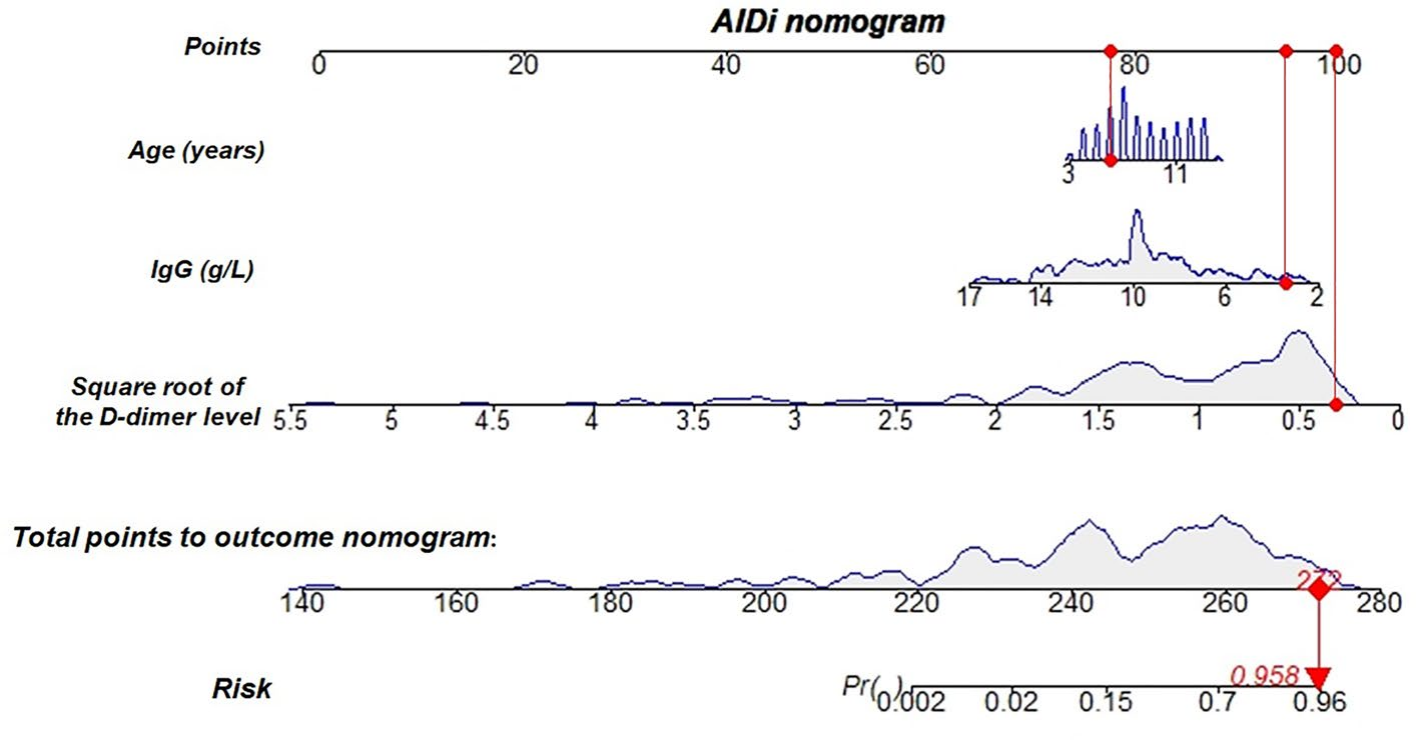
AIDi nomogram for predicting the risk of IgA vasculitis nephritis.

A nomogram of AIDi was developed (Fig. 3). The distribution of one patient of IgA vasculitis nephritis was shown to illustrate how to use the nomogram. The red dot at each line represented the value of each of the 3 predictors for this patient. The total points of this patient were 272, corresponding to a probability of 0.958 for the risk of IgA vasculitis nephritis.

### AIDi nomogram had a good performance in predicting the risk of IgA vasculitis nephritis with different histological grades and gender

**Figure 4:**
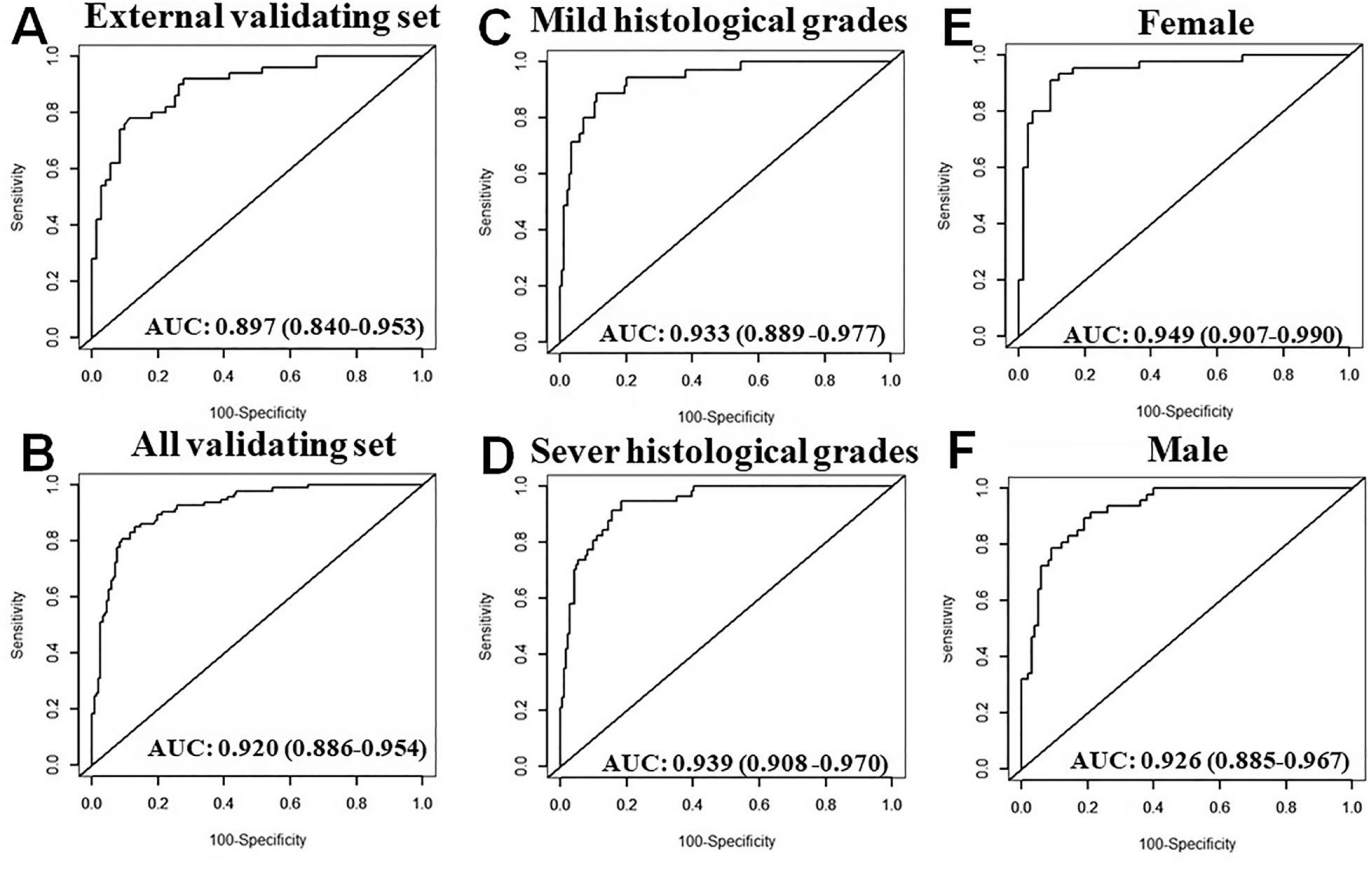
Receiver operating characteristic curve analysis of AIDi nomogram for predicting the risk of IgA vasculitis nephritis in different stratified sets. AUROC of the AIDi predicting the development of IgA vasculitis nephritis in external validating set (n = 122) (**A**) and in all validating set (internal and external validation, n = 250) (**B**); AUROC of AIDi predicting the risk of IgA vasculitis nephritis in the mild histological grades group (I and IIa, n = 35) (**C**) and the sever histological grades (IIb and above, n = 57) (**D**); AUROC of the AIDi predicting the risk of IgA vasculitis nephritis in females (n = 119) (**E**) and males (n = 147) (**F**).

AIDi nomogram was verified in an external validating set and all validating set (a combination of internal and external validating sets). As shown in Fig. 4A,B, the AUROC also showed a good predictive effect for the risk of IgA vasculitis nephritis (external validating set: 0.897, 95% CI: 0.840–0.953; all validating set: 0.920, 95% CI: 0.886–0.954) respectively.

To optimize the clinical application of AIDi nomogram, stratification analysis by gender and IgA vasculitis nephritis histological grades were performed using the ROC analysis. Histological grade of IgA vasculitis nephritis were stratified in two groups: mild (I and IIa) and sever histological grades (IIb and above). These AUROC values were 0.933 (95% CI: 0.889–0.977) (Fig. 4C) for mild histological grades subgroup, 0.939 (95% CI: 0.908–0.970) (Fig. 4D) for sever histological grades subgroup, 0.949 (95% CI: 0.907–0.990) (Fig. 4E) for the female and 0.926 (95% CI: 0.885–0.967) (Fig. 4F) for the male, respectively. Altogether, these results indicated that AIDi nomogram had a high predictive efficacy for all the patients regardless of gender and histological grads.

## Discussion

IgA vasculitis nephritis, a serious and long-term complication of IgA vasculitis, has been considered responsible for the prognosis of IgA vasculitis^13^. Early diagnosis and timely treatment of IgA vasculitis nephritis are crucial to improve the prognosis of IgA vasculitis patients. Therefore, there is an urgent need for a predictive model to help clinicians accurately distinguish the nephritis cases from IgA vasculitis patients. In this study, using a large case–control study cohort of 388 individuals, a simple predictive model AIDi for risk of nephritis in IgA vasculitis patients was constructed and assessed. The model AIDi was converted to nomogram for visual use for the first time. This model had high efficiency for predicting the risk of occurrence of nephritis in IgA vasculitis patients (AUROC: 0.920) regardless of gender and IgA vasculitis nephritis histological grads. Its efficiency was also validated in the external validation set with similar result (AUROC: 0.897).

Currently, nephritis is a manifestation of IgA vasculitis involving genetic and host susceptibility factors influencing humoral and cell-mediated immunity, cytokines, and the fibrinolytic-coagulation system^14,15^. Hence, a comprehensive model composed of parameters derived from demographic characteristics, clinical features, immune-inflammation and fibrinolytic-coagulation factors has potential to predict the risk of IgA vasculitis nephritis. As shown in our study, 8 candidate variables (age, weigh, duration of rash, PLT count, CRP, D-dimer, IgG and C3) were initially screened out in the univariate logistic analysis, and these variables belong to the categories mentioned above. Among these candidate variables, age and duration of rash have been identified as two key clinical features in predicting the IgA vasculitis nephritis^4,5,7^. In a large study including 535 patients, the researchers confirmed that older age at onset, or who have purpura on the upper limbs or face, or who have occult blood in the stool were particularly monitored for signs of IgA vasculitis nephritis^4^. Buscatti et al. also reported that persistent rash over a period longer than 1 month was a significant predictor of IgA vasculitis nephritis occurrence^5^. In our study, both age and duration of rash were also identified as independent risk factors for IgA vasculitis nephritis which was consistent with above studies, their combination in the predictive model can offer lots of potential advantages in the prediction of occurrence of IgA vasculitis nephritis.

In this study, D-dimer level was found to have the predictive power for occurrence of IgA vasculitis nephritis. D-dimer was regarded as one of the molecular markers for activity of fibrinolytic system, which is disordered in the whole process of IgA vasculitis^11,15^. It was reported that a significantly elevated level of D-dimer suggested a state of hyperfibrinolysis in the early stage of onset of IgA vasculitis^16,17^. In our study, comparing to the early stage of IgA vasculitis, we found that the level of D-dimer was significantly reduced in IgA vasculitis nephritis patients (1.88 (0.90, 3.55) vs. 0.32 (0.23, 0.51), *p* = 0.0001 in the training set). This may be due to the fact that with the remission of the IgA vasculitis, hyperfibrinolysis can also be alleviated and corrected by anticoagulant therapy^11^, thus leading to a significant reduction in the level of D-dimer, when there was occurrence of IgA vasculitis nephritis. However, the involved mechanisms are still not fully elucidated and deserve further research.

Our study also identified the significantly decreased level of serum IgG as a predictor for occurrence of IgA vasculitis nephritis. Compared to the level in IgA vasculitis patients, serum IgG level was significantly decreased in patients with IgA vasculitis nephritis (10.25 (8.69, 12.38) vs. 9.30 (6.11, 9.87), *p* = 0.001 in the training set), which indicated that there was humoral immune dysfunction throughout IgA vasculitis nephritis as reported in pervious study^9^. IgA vasculitis nephritis was considered to be caused by the glomerular deposition of immune complexes (including IgA1 and IgG) in the mesangium, the subepithelial and the subendothelial space^18,19^. The deposition and consumption of immune complexes may be contributed to the decrease of serum IgG level. In addition, the abnormal function of B cells in nephrotic syndromes was also involved with the decrease of serum IgG level. It was reported that low capacity of activation and proliferation of B cell inhibits the secretion and transformation of IgG and resulted in the decrease of serum IgG level^20^.

Among four independent risk factors, the risk factor “duration of rash” needs to be consulted from the guardian of the pediatric patients by the clinicians. Due to the limited knowledge of rash manifestation in IgA vasculitis, the guardians of pediatric patients can hardly recognize the rash and provide accurate duration of rash. Although the AIDD model (including all independent risk factors) also has the highest predictive efficiency in this study, the error in the duration of rash can significantly affects the predictive accuracy of the AIDD model in clinical practice. However, age, serum IgG level and serum D-dimer level can be accurately obtained from lab examination and demographic data by the clinicians. Therefore, using these three objective and accurate predictors, we constructed the AIDi model. After assessed by the ROC, calibration curve, DCA, NRI and IDI in validating set and stratification analysis, AIDi model can achieve the similar AUROC and predictive accuracy to the AIDD model.

With the routine intravenous blood sample collection, rapid measurement and accurate laboratory results, serum IgG and D-dimer level measurement are commonly available even in community clinics. AIDi nomogram provides the clinicians with a convenient and effective tool for assessing the risk of nephritis in IgA vasculitis patients. Actually, strengthening the longitudinal follow-up, such as repeated urinalyses at specific intervals, is a very effective way for early diagnosis of nephritis among IgA vasculitis patients. The application of AIDi nomogram is to improve the predictive accuracy of nephritis occurrence among IgA vasculitis patients in each longitudinal follow-up. Here the guideline of AIDi nomogram can be suggested: (i) the clinicians diagnose IgA vasculitis according to the diagnostic criteria; Meanwhile, the serum IgG and D-dimer level were also measured; (ii) as shown in Fig. 3, the total points were calculated by summing the points identified on the “Point” scale for each predictor. By comparing the “Total points” scale and the “Risk” scale, the individual risk of IgA vasculitis nephritis could be obtained; (iii) if the risk of the patient is greater than 0.7, the patients highly recommended for close follow-up care, whose follow-up were suggested to be shortened within 2 weeks or even 1 week and more detailed urine test items were required. Otherwise, they can receive routine follow-up care, who were suggested to be performed routine urinalysis within 4 weeks. Altogether, AIDi nomogram can be easily applied in clinical practice and help to discriminate patients with IgA vasculitis nephritis at an early stage, which may provide an opportunity for clinicians to optimize clinical treatment.

The present study has several limitations. Although a total of 386 participants from two medical centers were eventually enrolled for training and validation of the model, it also needs to be further validated as yet with large prospective studies from multicenter. Additionally, this model developed from Asian pediatric patients. Our result may not be representative of other race patients in general. We encourage our model to undergo validation in other races and also in centers outside China.

## Methods

### Participants

388 participants were enrolled in this retrospective study finally. Among these, 266 participants were collected from Shandong Provincial Hospital Affiliated to Shandong First Medical University between 2016.08 and 2021.04, 122 participants were collected from Children’s Hospital of Soochow University between 2017.06 and 2021.08. Ethics approvals from the Ethic committee of the Shandong Provincial Hospital Affiliated to Shandong First Medical University and the Ethic committee of the Children's Hospital of Soochow University were taken. Written informed consents were obtained from all the parents/legal guardians of all participants. All methods were performed in accordance with the relevant guidelines and regulations. The diagnosis of IgA vasculitis and IgA vasculitis nephritis were established according to EULAR/PRINTO/PRES criteria^21^. As far as this study is concerned, the patients, who are in the presence of purpura or petechiae (mandatory) with lower limb predominance and at least one of the following findings: abdominal pain or arthritis or arthralgia, are classified into IgA vasculitis group. Repeat urinalyses at specific intervals was performed during the follow-up of IgA vasculitis patients. If these IgA vasculitis patients are in the presence of hematuria and/or proteinuria within 6 months of IgA vasculitis onset, renal biopsy will be advised. If their renal histopathology were IgA nephropathy, they will be classified as IgA vasculitis nephritis group. Exclusion criteria were as follows: (1) serious complications of IgA vasculitis (e.g., intussusceptions, intestinal perforation, intracranial hemorrhage); (2) previous abnormal urine test or other diseases causing renal injury (e.g., primary IgA nephropathy, systemic lupus erythematosus, primary nephrotic syndrome, etc.); (3) receiving hormone or immunosuppressive treatment 3 months before admission; (4) contraindications of renal biopsy (e.g., solitary kidney, malformation of urinary system, trauma or history of renal surgery, etc.); (5) insufficient medical records. The histological grade of IgA vasculitis nephritis were assigned according to the International Study of Kidney Disease in Children classification^22^: grade I, minor glomerular abnormalities; grade IIa, focal segmental mesangial proliferation (MP) without crescents; grade IIb, diffuse MP without crescents; grade III, MP with < 50% crescents; grade IV, MP with 50% to 75% crescents; grade V, MP with > 75% crescents; grade VI, membranoproliferative-like lesions. All histological grade of renal biopsy were reviewed by a single pathologist and were stratified in two groups: mild (I and IIa) and sever histological grades (IIb and above)^23,24^.

### The pediatric patients’ enrollment, grouping and the study design

**Figure 5:**
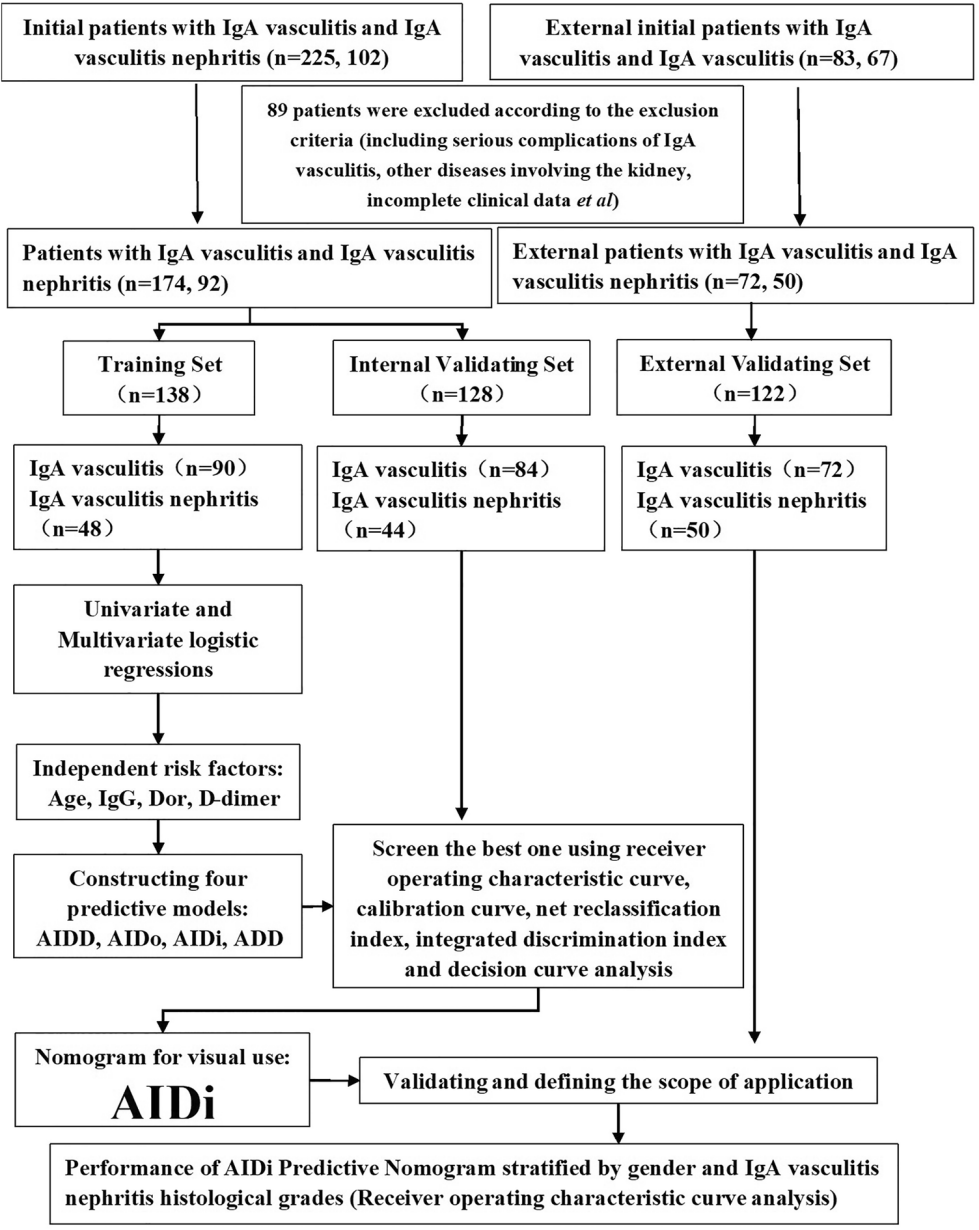
Flow chart of patients’ enrollment, grouping and the study design.

As shown in Fig. 5, 89 participants were excluded and 388 participants were finally enrolled in the analysis, including 174 IgA vasculitis patients, 92 IgA vasculitis nephritis patients and 122 patients in an external validating set. 266 patients were randomly divided into two sets: a training set (n = 138) including 90 IgA vasculitis patients and 48 IgA vasculitis nephritis patients, and the internal validating set (n = 128) including 84 IgA vasculitis patients and 44 IgA vasculitis nephritis patients, which were used to construct and validate the model respectively. The external validating set (including 72 IgA vasculitis patients and 50 IgA vasculitis nephritis patients) matching with patients by age and gender were used to further validate the nomogram’s predictive efficiency. All of the IgA vasculitis nephritis patients were confirmed by renal biopsy to have mesangial proliferative glomerulonephritis dominated by IgA mesangial deposition.

The following data were collected within 24 h at the time of admissions: demographic data, Dor, CBC with differential, immune globulins, coagulation and fibrinolytic system indicators, CRP and complement serum level. The term “duration of rash” was defined as the time of rash recurrent or persistent, and was dichotomized into within a month and more than a month category. Peripheral CBC with differential included WBC, LYM, MON, NEU count, RBC count and related information: HGB, MCV and RDW, PLT counts and related information: PDW and MPV. Immune globulin included IgA, IgM, and IgG. Coagulation and fibrinolytic system indicators included D-dimer and APTT. Serum complement included C3 and C4.

### Laboratory evaluation

All tests performance and results verification were conducted in Shandong Provincial Hospital Affiliated to Shandong First Medical University and Children's Hospital of Soochow University. Peripheral CBC, immune globulins, coagulation and fibrinolytic system indicators, CRP, and serum complements were measured in samples from the peripheral vein blood samples. Peripheral CBC and CRP was determined using an automated MEK-8222K hematology analyzer (Nihon Kohden, Japan). Immune globulins and complements were performed using the fully automatic specific protein analyzer A25 (BioSystems, Spain). Coagulation indicators were performed using an automatic coagulation analyzer CS5100 (SYSMEX, Japan).

### Statistical analysis

Data were expressed as mean ± SD (or median, IQR) for quantitative variables and percentages for qualitative variables. The statistical significance between two groups was determined by *t* test or rank sum test for continuous variables and Chi-square test or Fisher’s exact test for categorical variables. The only endpoint in this study is the diagnosis of IgA vasculitis nephritis. Univariate logistic analysis was firstly performed on all variables between patients with and without the study endpoint in training set. All selected variables were analyzed by multivariate logistic analysis to assess the independent risk factors and were used to construct the predictive model. By reducing the predictors of the model, a series of simplified model were derived. Predictive efficiency of these models was evaluated using the receiver operating characteristic curve (ROC) and verified in internal and external validating set. Calibration curve and decision curve analysis (DCA) were performed to evaluate the accuracy and clinical effectiveness of these models. The net reclassification index (NRI) and integrated discrimination index (IDI) were calculated to further compare the predictive efficiency between these models^25^. A nomogram was preformed based on fitted logistic regression model and by the R package *Regression Modeling Strategies* (*rms*) in R version^26^. Stratification analysis of IgA vasculitis nephritis histological grades and gender was used to seek the optimum application scope of the predictive model. All statistical analyses were performed using R software version 3.4.3 (http://www.rproject.org/). Two-tailed *p* < 0.05 was considered statistically significant.

## Supplementary Information

Supplementary Figure S1.

Supplementary Figure S2.

Supplementary Table S1.

Supplementary Legends.

## Data Availability

The datasets generated and analyzed during the current study are available from the corresponding authors on reasonable request.
