# Supplementary figures and images for: A simple nomogram for assessing the risk of IgA vasculitis nephritis in IgA vasculitis Asian pediatric patients
Source: Sci Rep. 2022 Oct 7;12:16809. doi: 10.1038/s41598-022-20369-3 (PMC9547060; doi:10.1038/s41598-022-20369-3)

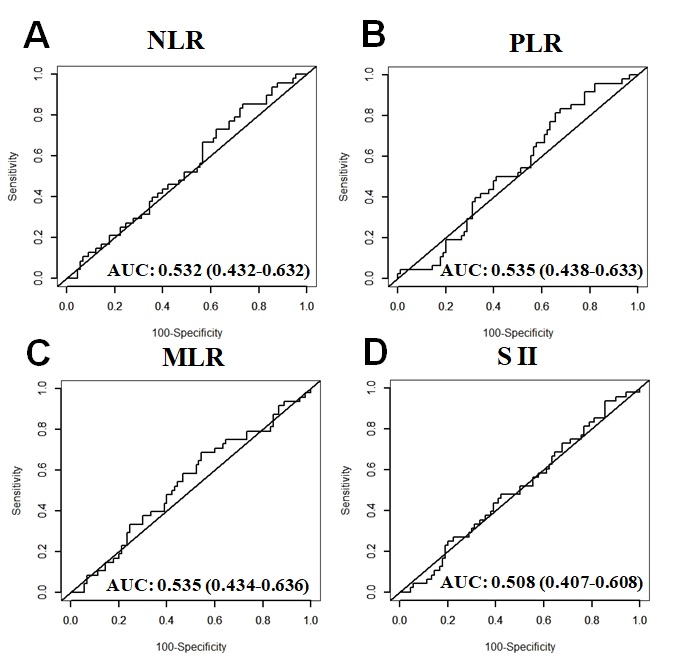

Supplement: Supplementary file 1 — Supplementary Figure S1. [file 41598_2022_20369_MOESM1_ESM.jpg]

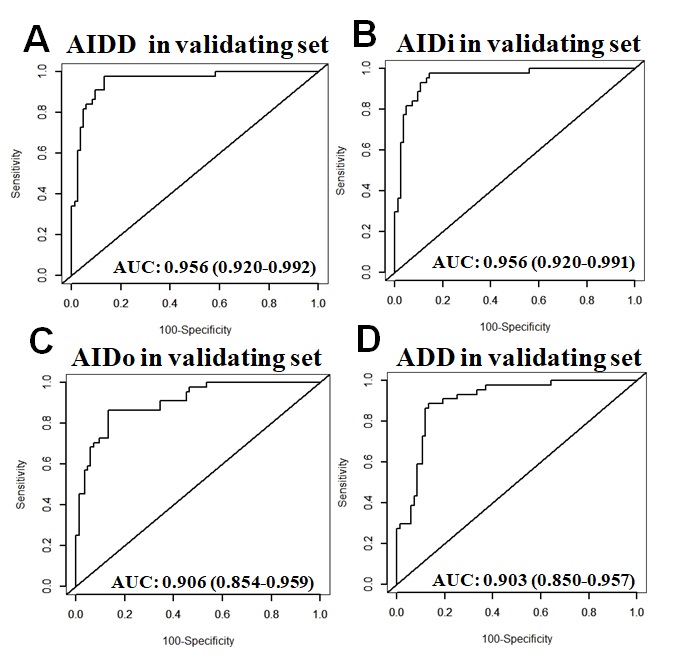

Supplement: Supplementary file 2 — Supplementary Figure S2. [file 41598_2022_20369_MOESM2_ESM.jpg]
