# Supplementary material for: A simple nomogram for assessing the risk of IgA vasculitis nephritis in IgA vasculitis Asian pediatric patients
Source: Sci Rep. 2022 Oct 7;12:16809. doi: 10.1038/s41598-022-20369-3 (PMC9547060; doi:10.1038/s41598-022-20369-3)
Supplement: Supplementary file 3 — Supplementary Table S1. [file 41598_2022_20369_MOESM3_ESM.doc]

Table S1. Characteristics of pediatric with IgA vasculitis and IgA vasculitis nephritis in external set.

| Variables | IgA vasculitis  (n= 72) | IgA vasculitis nephritis  (n = 50) |
| --- | --- | --- |
| Gender  Female, n (%) | 35 (48.6) | 19(38.0) |
| Male, n (%) | 37 (51.4) | 31(62.0) |
| Age, IQR (years) | 7.00(6.00,9.00) | 9.00(8.00,11.75) |
| Weigh, IQR (kg)  Duration of rash^#^ | - | - |
| Within a month, n (%) | 65 (90.3) | 47 (94.0) |
| More than a month, n (%) | 7(9.7) | 3 (6.0) |
| D-dimer (IQR) (mg/L) | 1.15(0.36, 2.01) | 0.54(0.29, 0.84) |
| IgG (IQR) (g/L) | 10.20(8.23, 11.52) | 8.30(6.46, 10.18) |

^#^Means the time of rash recurrent or persistent
