# Supplementary material for: A simple nomogram for assessing the risk of IgA vasculitis nephritis in IgA vasculitis Asian pediatric patients
Source: Sci Rep. 2022 Oct 7;12:16809. doi: 10.1038/s41598-022-20369-3 (PMC9547060; doi:10.1038/s41598-022-20369-3)
Supplement: Supplementary file 4 — Supplementary Legends. [file 41598_2022_20369_MOESM4_ESM.docx]

**Fig.S1 Receiver operating characteristic curve analysis of some predictive models of inflammation for predicting the risk of IgA vasculitis nephritis.** AUROC of NLR (A), PLR (B), MLR (C) and SⅡ (D)

**Fig.S2 Receiver operating characteristic curve analysis of 4 combined models for predicting the risk of IgA vasculitis nephritis in validating set.** AUROC of AIDD (A), AIDi (B), AIDo (C) and ADD (D).

**Table S1. Characteristics of pediatric with IgA vasculitis and IgA vasculitis nephritis in external set.**
